# Identification of 56 Proteins Involved in Embryo–Maternal Interactions in the Bovine Oviduct

**DOI:** 10.3390/ijms21020466

**Published:** 2020-01-11

**Authors:** Charles Banliat, Guillaume Tsikis, Valérie Labas, Ana-Paula Teixeira-Gomes, Emmanuelle Com, Régis Lavigne, Charles Pineau, Benoit Guyonnet, Pascal Mermillod, Marie Saint-Dizier

**Affiliations:** 1 INRAE, CNRS, Université de Tours, IFCE, UMR PRC, 37380 Nouzilly, France; charles.banliat@inra.fr (C.B.); guillaume.tsikis@inra.fr (G.T.); valerie.labas@inra.fr (V.L.); pascal.mermillod@inra.fr (P.M.); 2 Union Evolution, 35530 Noyal-sur-Vilaine, France; benoit.guyonnet@evolution-xy.fr; 3 INRAE, Université de Tours, CHU de Tours, Plate-forme CIRE, PAIB, 37380 Nouzilly, France; ana-paula.teixeira@inra.fr; 4 INRAE, UMR 1282 ISP, 37380 Nouzilly, France; 5 Inserm, University of Rennes, EHESP, Irset (Institut de recherche en santé, environnement et travail)—UMR_S 1085, 35000 Rennes, France; emmanuelle.com@univ-rennes1.fr (E.C.); regis.lavigne@univ-rennes1.fr (R.L.); charles.pineau@univ-rennes1.fr (C.P.); 6 Protim, Inserm U1085, Irset, Campus de Beaulieu, University of Rennes 1, Proteomics Core Facility, 35000 Rennes, France; 7 Faculty of Sciences and Techniques, Department Agrosciences, University of Tours, 37000 Tours, France

**Keywords:** tubal fluid, fallopian tube, oviduct, embryo, bovine, cattle, proteomics, secretions, morula, 4–6 cell

## Abstract

The bovine embryo develops in contact with the oviductal fluid (OF) during the first 4–5 days of pregnancy. The aim of this study was to decipher the protein interactions occurring between the developing embryo and surrounding OF. In-vitro produced 4–6 cell and morula embryos were incubated or not (controls) in post-ovulatory OF (OF-treated embryos) and proteins were then analyzed and quantified by high resolution mass spectrometry (MS) in both embryo groups and in OF. A comparative analysis of MS data allowed the identification and quantification of 56 embryo-interacting proteins originated from the OF, including oviductin (OVGP1) and several annexins (ANXA1, ANXA2, ANXA4) as the most abundant ones. Some embryo-interacting proteins were developmental stage-specific, showing a modulating role of the embryo in protein interactions. Three interacting proteins (OVGP1, ANXA1 and PYGL) were immunolocalized in the perivitelline space and in blastomeres, showing that OF proteins were able to cross the zona pellucida and be taken up by the embryo. Interacting proteins were involved in a wide range of functions, among which metabolism and cellular processes were predominant. This study identified for the first time a high number of oviductal embryo-interacting proteins, paving the way for further targeted studies of proteins potentially involved in the establishment of pregnancy in cattle.

## 1. Introduction

In mammals, embryo development starts in the oviduct, a tubular organ connecting the ovary to the uterus. The bovine embryo develops up to the 16-cell or early morula stage in the oviduct, in close contact with the oviductal epithelial cells and their secretions, the oviductal fluid (OF) [1]. Important embryonic changes including the first mitotic cleavages and the embryonic genome activation, at 8-cell, occur in this oviductal micro-environment [2,3]. The OF is a dynamic and complex fluid composed of glycosaminoglycans, lipids, small metabolites, inorganic salts and a high number of proteins [4]. Recently, mass spectrometry (MS) techniques have considerably increased our knowledge of the OF proteomic composition in cattle [5,6,7]. Furthermore, extracellular vesicles (EVs) originating from the oviduct epithelium have recently been identified as major components of the OF [8]. The molecular cargo of bovine oviductal EVs, including proteins, mRNA and different types of non-coding RNA including microRNA, was reported to dynamically change across the estrous cycle [9,10]. While embryos can be produced in vitro in the absence of the oviductal micro-environment, there is evidence that the OF plays important roles in early embryo development. Embryos developed in isolated bovine or ovine oviducts are of higher quality than their in-vitro counterparts in terms of morphology, gene expression, cryotolerance and pregnancy rates after transfer into recipients [11,12,13]. In the same way, in vitro development with low concentrations of OF has been shown to improve the cryotolerance and gene expression of bovine blastocysts [14]. In particular, the presence of OF in the culture medium provided a better control of cattle embryo DNA methylation [14,15]. Furthermore, the addition of oviductal EVs to the culture medium increased blastocyst rate, extended embryo survival and improved the quality of in vitro-produced cattle embryos [9,16].

The proteins present in the OF, either within EVs or as soluble molecules, may play key roles in supporting embryo development. Indeed, the addition of purified or recombinant oviductin, an oviduct-specific estrus-induced glycoprotein, to the culture medium was reported to increase the rates of blastocyst development of goat [17] and cattle [18] embryos compared with non-supplemented controls. In another study, the presence of porcine recombinant oviductin did not enhance in vitro developmental rates but produced bovine blastocysts of higher quality regarding the expression of specific genes [19]. By immunostaining, oviductin has been shown to associate with oocytes and embryos from numerous species including the golden hamster [20,21], baboon [21,22], pig [23] and cattle [24,25]. Microscopic observation of in-vivo retrieved embryos revealed that oviductin crossed the zona pellucida and localized in the perivitelline space and inside embryo blastomeres [22,23,24,26,27]. Apart from oviductin, very few oviductal proteins have been identified as interacting with the early embryo. Co-incubation of bovine oocytes with biotinylated OF allowed researchers to identify six oviductal proteins of molecular masses ranging from 30 to 95 kDa associated with the zona pellucida [28]. However, the proteins other than oviductin, at 95 kDa, remained unknown. In another study that used immunostaining and western blot on bovine oocytes pre-treated with OF, three proteins, including oviductin, osteopontin (SPP1) and lipocalin-type prostaglandin D synthase (l-PGDS), were shown to associate with the zona pellucida [29]. Up to now, there is no information about additional oviductal proteins that may interact with the cattle embryo. Furthermore, whether the embryo–oviduct molecular interactions vary according to the developmental stage of the embryo is currently not known.

The aim of this study was to identify with no a priori OF proteins interacting with the bovine embryo at the 4–6 cell and early morula stages, two oviductal stages before and after embryo genomic activation. A MS-based approach was applied to more than 400 in vitro-produced embryos, allowing the identification of new embryo-interacting proteins, some of which being stage-specific. Immunostaining confirmed that the interacting proteins were able to cross the zona pellucida and be internalized by embryos.

## 2. Results

### 2.1. New Embryo-Interacting Proteins Were Identified by NanoLC-MS/MS and Changed According to the Embryonic Stage

Among the proteins identified in the OF, 56 were classified as interacting with embryos (i.e., detected in OF-treated embryos but not detected in controls, or detected at significantly higher abundance in OF-treated than in control embryos). The 56 embryo-interacting proteins accounted for only 0.03% of the 1707 proteins identified in the OF (see all OF proteins identified in Table S1).

In total, 4–6 cells and morulas interacted with 37 and 43 proteins, respectively (Table 1, Table 2 and Table 3), Interacting proteins accounted for 0.02% of the 1616 and 1765 proteins identified in OF-treated 4–6 cells and morulas, respectively (see all proteins identified in embryos in Table S2). The embryo-interacting proteins changed according to the developmental stage:

- 13 proteins interacted exclusively with 4–6 cells (Table 1).
- 19 proteins interacted exclusively with morulas (Table 2).
- 24 proteins interacted with both embryonic stages (Table 3).

There was no correlation between the initial abundance of the embryo-interacting proteins in the OF and their abundance in OF-treated embryos (Figure S1). Nineteen 4–6 cell-interacting proteins were detected only in OF-treated embryos and 18 were measured at a higher abundance in the OF-treated than in the control embryos (mean ± SEM of treated:control ratio = 8.1 ± 2.1; range: 2.1–37; Table 1 and Table 3). For morulas, 11 proteins were detected only in OF-treated embryos and 32 measured at higher abundance in OF-treated than in controls (mean ± SEM of treated:control ratio = 9.3 ± 2.7; range: 2–71; Table 1 and Table 2).

**Figure 1:**
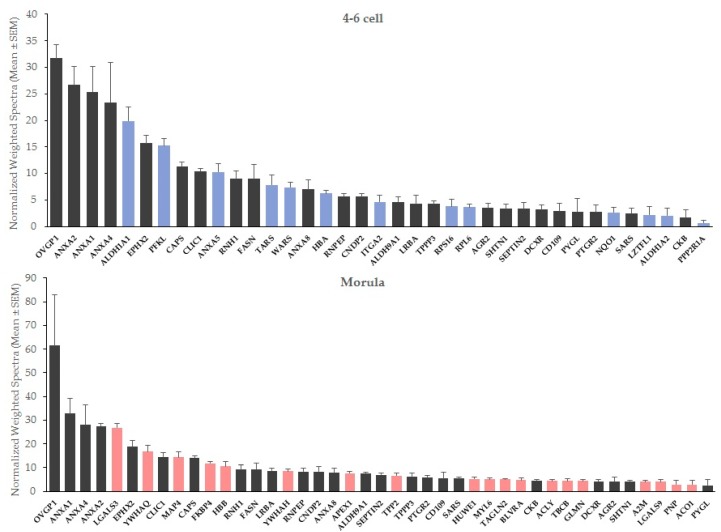
Mean abundance of embryo-interacting proteins in OF-treated embryos at the 4–6 cell and morula stages. Blue bars, proteins interacting exclusively with 4–6 cell embryos. Red bars, proteins interacting exclusively with morulas.

At both stages, oviductin (OVGP1) and annexins A1 (ANXA1), A2 (ANXA2) and A4 (ANXA4) were the most abundant interacting proteins in OF-treated embryos, followed in 4–6 cells by retinal dehydrogenase 1 (ALDH1A1), epoxide hydrolase 2 (EPHX2) and ATP-dependent 6-phosphofructokinase (PFKL), and for morulas, by galectin-3 (LGALS3), epoxide hydrolase 2 (EPHX2) and 14-3-3 protein theta (YWHAQ; Figure 1).

### 2.2. Most Embryo-Interacting Proteins Were Presumed to be Exosomal and Secreted via Non-Conventional Pathways

**Figure 2:**
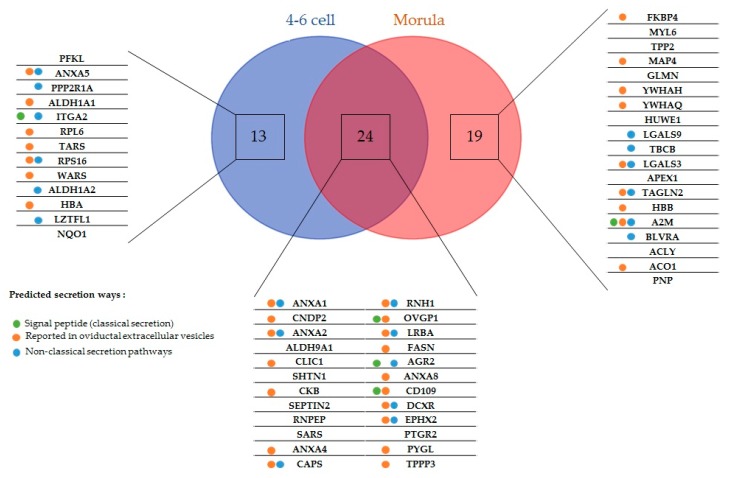
Embryo-interacting proteins classified according to the embryonic stage and secretion pathway. Colored spots indicate the presumed secretion pathways in oviduct epithelial cells tools. Green, proteins possessing a peptide signal and presumed to be conventionally secreted; blue, proteins predicted to be non-conventionally secreted; orange, proteins reported in bovine [9] and feline [30] oviductal exosomes.

Five embryo-interacting proteins (9%) contained a signal peptide and were predicted to be secreted in a conventional way. Furthermore, 20 (36%) were predicted to be secreted by non-classical pathways. In addition, 33 embryo-interacting proteins (59%) were reported previously in oviductal exosomes (Figure 2) [9,30].

### 2.3. Embryo-Interacting Proteins Were Mainly Involved in Metabolism and Cellular Processes

**Figure 3:**
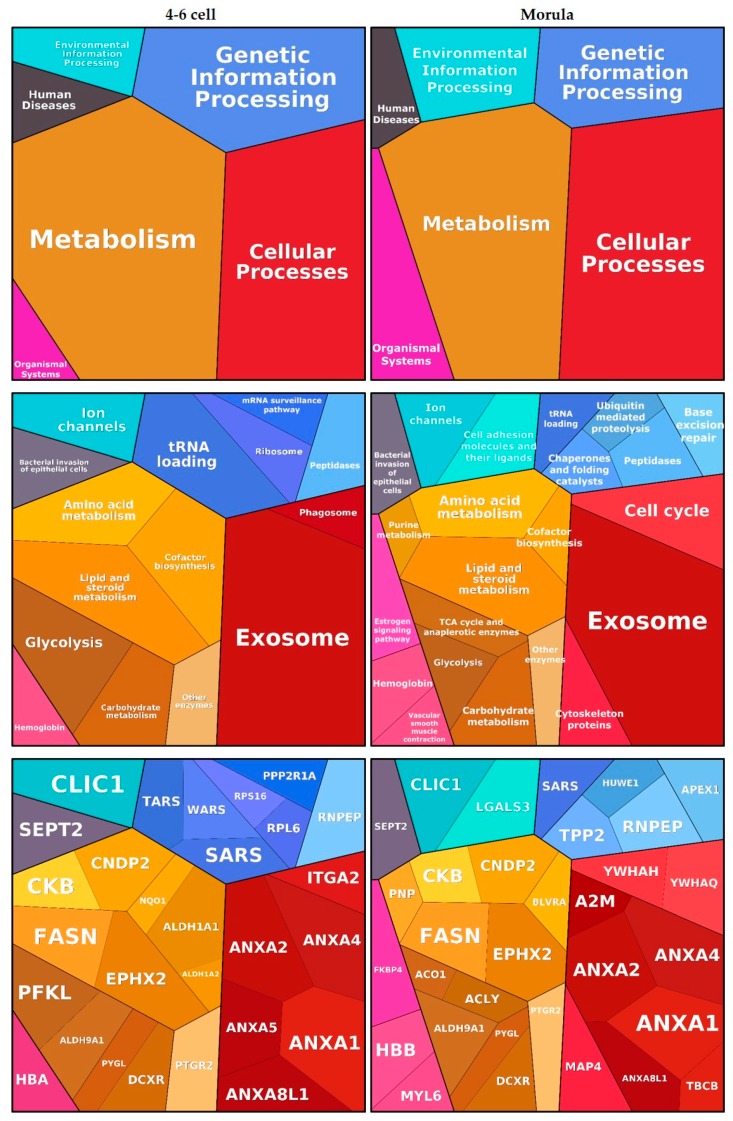
Functions associated with embryo-interacting proteins at the 4–6 cell and morula stages. The figures were automatically built in Proteomaps based on the KEGG (Kyoto Encyclopedia of Genes and Genomes) Pathway gene classification. Functional categories (up and middle panels) and related proteins (down panel) are shown by polygons. Areas of polygons illustrate protein abundance, weighted by protein size. Functionally related function/protein are arranged in common regions and coded using similar colors.

Functional annotation clustering of embryo-interacting proteins resulted in six enriched clusters, among which ‘Metabolism’ and ‘Cellular processes’ were the most significant. These findings are visualized using Proteomaps in Figure 3. Proteins such as annexins and alpha-2 macroglobulin (A2M) were assigned to the ‘Exosome’ category while proteins such as epoxide hydrolase 2 (EPHX2), retinal deshydrogenase 1 (ALDH1A1) and PYGL were assigned to various metabolic processes. Oviductin did not have any functional category annotation and therefore was not included in the proteomap.

### 2.4. Protein Interactions Were Localized in Different Embryo Subcompartments

**Figure 4:**
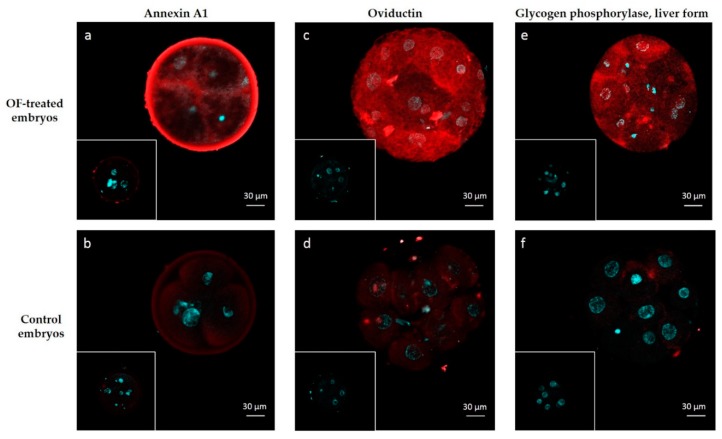
Immunolocalization of embryo-interacting proteins in OF-treated embryos (**a**,**c**,**e**) and controls (**b**,**d**,**f**). Proteins of interest are in red and nuclei are in blue. Embryos were incubated with anti-ANXA1 (**a**,**b**), anti-OVGP1 (**c**,**d**) and anti-PYGL (**d**,**e**). Inserts: controls with isotypes instead of primary antibodies.

Two highly abundant interacting proteins (OVGP1 and ANXA1) and one among the least abundant ones (PYGL) were chosen to visualize protein interactions in 4–8 cell embryos by immunohistochemistry. The signal for ANXA1 was recorded in the zona pellucida, perivitelline space and into blastomeres of OF-treated embryos (Figure 4a). The pattern of interactions was slightly different for OVGP1 and PYGL, which was localized in the perivitelline space and in blastomeres but not in the zona pellucida of OF-treated embryos (Figure 4c,e, respectively). A negligible diffuse signal was observed in blastomeres of control embryos incubated with primary antibodies against ANXA1, OVGP1 and PYGL (Figure 4b,d,f). No signal was detected in OF-treated and control embryos incubated with IgG isotypes (frames in Figure 4).

## 3. Discussion

The bovine embryo develops in contact with the OF for the first 4–5 days of its life. To date, there is limited information regarding the molecular interactions occurring between the embryo and its maternal microenvironment. In this study, using a high-resolution MS technique, a number of new embryo-interacting proteins originated in the OF were identified. To our knowledge, this is the first study providing a significant list of proteins interacting with the early embryo in mammals.

The first criterion retained for the definition of embryo-interacting proteins was their detection in the post-ovulatory OF ipsilateral to ovulation. A total of 1707 proteins were identified by nanoLC-MS/MS in the OF used to produce OF-treated embryos. The most abundant proteins in the OF included serum albumin, heat shock proteins (HSP90AA1, HSP90B1, HSP90AB1, HSPA1B, HSPA5), oviductin (OVGP1), annexin A4 (ANXA4), complement C3 (C3), myosin 9 (MYH9) and numerous tubulin subunits. This is in agreement with previous proteomic analyses of post-ovulatory OF collected from cows at the slaughterhouse [5] or by transvaginal endoscopy [7]. Based on our previous work on the regulation of the bovine OF proteome across the estrous cycle [5], some proteins more abundant in the OF around the time of ovulation compared with the luteal phase were identified as embryo-interacting proteins. This is the case, among others, for OVGP1, CD109 and PFKL. Some of these proteins and others were also at higher levels on the side of ovulation, i.e., the side of embryo development, than on the contralateral side at the post-ovulatory stage—this is the case for A2M, CD109 and PFKL [5]. However, at the same stage, EPHX2 was less abundant in the ipsilateral than in the contralateral OF, showing that the secretion of some but not all embryo-interacting proteins may be upregulated at the time and place of embryo presence in the oviduct.

We hypothesized that numerous proteins present in the OF could interact with in vitro- produced early embryos. However, the 56 embryo-interacting proteins accounted for only 0.03% of the identified OF proteins. To our knowledge, this is the first study deciphering OF embryo-interacting proteins using a MS-based approach with no a priori. In an earlier study using the same methodology on bovine spermatozoa, we identified 27 oviductal proteins that interacted with bovine sperm cells [31]. Similarly, sperm-interacting proteins accounted for less than 0.06% of proteins previously identified by MS in the OF [5,31]. In addition to the low proportion of embryo-interacting proteins among OF proteins, there was no relationship between the initial abundance of the embryo-interacting proteins in the OF and their abundance in OF-treated embryos. To illustrate this, galectin-3 (LGALS3) was the fifth most abundant embryo-interacting protein in morulas but was detected with low abundance (12 normalized weighted spectra (NWS)) in the OF. On the other hand, PYGL was among the top-50 most abundant proteins (83 NWS) in the OF but one of the least abundant embryo-interacting proteins. Thus, it seems that very few OF proteins interacted with embryos and that these interactions were not related to their initial abundance around embryos, suggesting highly selective and specific embryo-OF interactions. However, we cannot exclude that longer incubation times (>6 h) may enable more OF proteins to interact. Moreover, this study was carried out on in-vitro produced embryos for obvious economical and ethical reasons (more than 800 embryos were used). Although oviductin is known to interact with bovine embryos in vivo [24] and was identified as interacting proteins under our conditions, it cannot be ruled out that embryo–protein interactions differ in vivo.

Oviductin was identified by nanoLC-MS/MS as interacting in high abundance with both 4–6 cells and morulas and was immunolocalized in the perivitelline space and blastomeres of OF-treated embryos. This is in agreement with previous studies in which OVGP1 was identified by immunostaining and/or western blot in bovine oocytes [28,29] and embryos [19,24] exposed to OF in vivo or in vitro. However, we did not observe a strong signal for OVGP1 in the zona pellucida of OF-treated embryos. Bovine embryos collected in vivo [24] or produced in vitro in the presence of recombinant OVGP1 [19] displayed high immunostaining in the zona pellucida. ANXA1 was observed in the zona pellucida of OF-treated embryos, showing that our conditions did not prevent protein interactions with the zona pellucida. These differences in OVGP1 localization may be due to differences in the origin of embryos used and to the duration of contact with OF or recombinant OVGP1 before immunostaining. A 6-h incubation was used in the present study, whereas embryos were retrieved in vivo after approximately 2 days within the oviduct in the study from Boice et al. [24] or incubated in vitro with recombinant OVGP1 for 3.5 days in the study from Algarra et al. [19]. These differences may also be due to the different antibodies used: a monoclonal antibody raised against the C-terminus of mouse oviductin in the present study vs. a home-made polyclonal antiserum directed against bovine oviduct glycoproteins [24], or a home-made monoclonal antibody directed against purified recombinant porcine OVGP1 [19].

In addition to oviductin, osteopontin (SPP1) and L-PGDS have been reported earlier as oviductal proteins interacting with the zona pellucida of bovine oocytes [29]. However, in this study, SPP1 and l-PGDS were not identified in the OF used for embryo incubation, and therefore could not be identified as interacting proteins. In line with our results, SPP1 and L-PGDS were not identified in previous MS-based analyses of bovine OF, either throughout the estrous cycle [5] or at Days 1 and 3 of the estrous cycle [7], both studies identifying more than 3000 proteins. Thus, the presence of SPP1 and L-PGDS in the bovine OF and their potential interaction with the bovine embryo cannot be confirmed. Moreover, inactivated complement-3b (iC3b), a derivative of the human complement protein C3 (C3), was shown to be taken up by mouse embryos, resulting in an increase in embryo development up to the blastocyst stage [32]. In the present study, C3 was identified at high abundance in the post-ovulatory OF (113 NWS, Table S1). However, C3 was not identified as interacting with cattle embryos. Therefore, some oviductal protein interactions with embryo are likely to be species-specific.

There is some evidence that the developing embryo interacts with its oviductal microenvironment [13,33,34]. However, little is known about the modulating role played by the embryo in these interactions. In order to address this question, the same OF and conditions of embryo incubation were used for both 4–6 cells and morulas. The results showed that 13 OF proteins interacted exclusively with 4–6 cell embryos, while 19 interacted only with morulas. Furthermore, for some proteins interacting at both stages, their abundance in OF-treated embryos differed between stages. For instance, the fold-change between OF-treated and control embryos for ANXA1 was approximately twice higher in 4–6 cells than in morulas (37 vs. 15). This suggests a modulating role played by the zona pellucida and/or embryonic cells in the process of protein interaction. The zona pellucida surrounding all mammalian embryos constitutes the first barrier for interactions between OF protein and embryonic cells. Several studies indicated that the zona pellucida is a dynamic envelope that changes in structure and properties depending on its environment [35,36,37]. The zona pellucida of mouse oocytes were shown to be permeable to macromolecules at molecular weights up to 170 kDa, while zygotes showed a decreased permeability at around 110 kDa [37]. Using colored molecular probes, it was shown that the size and hydrophilic–lipophilic balance of the probe were important in determining its interaction with the mouse embryo [36]. Scanning electron microscopy observation of bovine in vitro-produced embryos showed that the outer zona pellucida surface typically forms a spongy network with a rough surface containing numerous pores; however, the mean number of pores doubled from the 8-cell to the morula stages (1658 vs. 3259 per 5000 µm^2^) and their mean diameter decreased in parallel (203 vs. 155 nm) [35]. These changes may contribute to the observed stage-specific embryo-protein interactions. Furthermore, the 8-cell stage was identified as the period of major embryonic genome activation in the bovine embryo [3,38]. At this time, maternal RNAs and proteins stored in the oocyte are gradually degraded and actively replaced by embryonic transcripts and proteins [3]. Therefore, molecules and interaction processes at the embryonic cell surface are likely to change from 4–6 cell to morula and to contribute, in association with changes in the zona pellucida permeability, to the differences in OF protein interactions between embryonic stages.

Oviductal EVs comprise exosomes, which are small 30–150 nm vesicles endocytic in origin and released upon fusion of multi-vesicular bodies with the membrane of oviduct epithelial cells, and microvesicles, which are larger vesicles (100–1000 nm) budding directly from the cell membrane [8]. The proteomic contents of oviductal EVs were recently published in the bovine [9] and feline [30], with many more proteins identified in the latter (1511 vs. 319 protein groups). Therefore, both species were considered to analyze potential secretion pathways of interacting proteins. In the present study, only five embryo-interacting proteins (9%) contained a signal peptide and appeared likely to be secreted in a classical way, whereas the majority of proteins were presumed to be secreted by non-conventional pathways and/or previously reported in oviductal EVs, including OVGP1, several annexins (A1, A2, A4, A5) and the liver form of glycogen phosphorylase (PYGL). It is important to note that these secretion pathways are not exclusive—interacting proteins like OVGP1, CD109 and alpha-2 macroglobuline (A2M) possess a signal peptide but were also identified in oviductal EVs. Furthermore, in the present study, OVGP1, ANXA1 and PYGL were immunolocalized in the perivitelline space but also in blastomeres of OF-treated embryos, showing that these molecules crossed the zona pellucida and were internalized by embryonic cells. To our knowledge, this is the first report of immunolocalization of ANXA1 and PYGL in mammalian embryos. Bovine embryos were shown to be able to internalize PKH67-labelled in vivo-derived oviductal EVs during in vitro development [9]. Cloned and parthenogenic porcine embryos were also able to uptake embryo-derived membrane-labelled EVs from the culture medium [39]. In both studies, oviductal EVs were observed in the whole cytoplasm of blastomeres [9,39]. Using electron microscopy on 2- to 8-cell embryos collected from hamster oviducts, endocytic structures, many endosomes and multivesicular bodies associated with OVGP1 immunolabeling were observed in the blastomeres [26]. Taken together, these results strongly suggest that oviductal proteins previously reported as exosomal were internalized into OF-treated embryos via exosomal cargos. However, the exact mechanisms by which OF proteins interacted with embryonic cells were beyond the scope of this study and remain to be determined.

Cattle embryos enter the uterus at the early morula stage and have a long pre-implantation period during which blastocyst hatching (Days 9–10), trophoblast elongation and intense production of interferon-tau (IFNT) occur before implantation begins, around Day 19 of pregnancy [2]. The possible functions of oviductal interacting proteins on pre-implantation steps are poorly understood. Some roles played by oviductin on early embryo development were reported from in vitro studies. Consistent positive effects of purified oviductin were observed on blastocyst yield in goat [17], sheep [40], pig [41] and cattle [18]. Antibodies directed against the C-terminal peptide of rabbit oviductin were shown to inhibit mouse embryo development at the 2-cell stage, suggesting that in this species, oviductin has a function in overcoming the development block at this stage [42]. In bovine, the addition of porcine recombinant oviductin during in vitro fertilization, in vitro development or both, increased the relative abundance in the embryo of mRNA of *DSC2*, *ATF4*, *AQP3* and *DNMT3A*, genes involved in cell proliferation, cell adhesion, cellular homeostasis and epigenetics [19].

Four annexins, namely ANXA1, ANXA2, ANXA4 and ANXA5, were identified as embryo-interacting proteins, only at the 4–6-cell stage for ANXA5 and at both stages for ANXA1, ANXA2 and ANXA4. It is well established that some annexins, including ANXA1 and ANXA2, can be secreted out of the cell through unconventional secretory mechanisms, with implications in many functions such as the endocrine regulation, inflammatory response and cancer [43]. Several studies have associated annexins with early embryo–maternal interactions. A greater abundance of ANXA4 was reported in the OF of pregnant mares compared with cyclic mares four days after ovulation [44] and both ANXA1 and ANXA2 were increased in the uterine fluid around the signaling of maternal recognition in this species [45]. Similarly, increasing amounts of ANXA1, ANXA2 and ANXA5 were reported in the uterine fluid of pregnant ewes in the pre-implantation period [45]. Annexin A1 knock-out female mice displayed numerous changes in early gestation, including increased sites of implantation, increased inflammatory reaction in the uterine fluid during implantation, reduced pre- and post-implantation losses and enhanced plasma progesterone [46]. Furthermore, ANXA2 was shown to be crucial for embryo adhesiveness to the endometrium, a critical step for implantation, in humans [47] and mice [48].

Galectin-3 and -9 interacted only with morulas and galectin-3 was one of the most abundant interacting proteins at this stage. Galectins have a varied array of activities both inside and outside cells [49]. Galectin-3 and -9 are members of the lectin family and contain carbohydrate recognition domains [49]. Galectin-3 is expressed in several parts of the female genital tract, including the uterine endometrium and oviduct [50,51]. When galectin-3 was knocked down in the mouse endometrium, the number of embryos implanted decreased substantially [51], showing that, like annexins, galectins have important roles in the establishment of pregnancy in mice.

In conclusion, proteins in the post-ovulatory OF that interact with the early bovine embryo before and after the embryonic genome activation were identified and quantified on a large scale for the first time. Some protein interactions were developmental stage-specific, revealing new roles of the embryo in modulating early maternal interactions. These data provide new protein candidates potentially involved in pre-implantation development and establishment of pregnancy in cattle. Targeted studies are required to go further in the search for underlying mechanisms and functions.

## 4. Materials and Methods

All reagents were purchased from Sigma-Merck (Saint-Louis, MO, USA) if not otherwise stated. PMSG, hCG and PG-600 were obtained from MSD Animal Health (Brussels, Belgium). Bovine trypsin was obtained from Roche Diagnostics GmbH (Basel, Switzerland). Paraformaldehyde (sc-281692) and mouse monoclonal antibodies raised against OVGP1 (sc-377267) and PYGL (sc-517597) were obtained from Santa-Cruz Biotechnology (Dallas, TX, USA). Goat polyclonal antibody raised against ANXA1 (AP22515PU-N) was obtained from Origene (Rockville, MD, USA). Secondary antibodies coupled with Alexa Fluor 633 (A21050 for ANXA1; A21082 for OVGP1 and PYGL) were obtained from Invitrogen Molecular Probes (Eugene, OR, USA).

### 4.1. Bovine Oviductal Fluid (OF) Collection

Oviducts connected to ovaries from adult *Bos taurus* cows were collected at a slaughterhouse and transported to the laboratory on ice within 2 h after the death of the animal. According to the morphology of the ovary and corpus luteum, only oviducts ipsilateral to the side of ovulation at the post-ovulatory phase of the estrous cycle (Days 1–5, i.e., at the expected time and place of embryo development) were used. Mixtures of OF and epithelial cells were collected from the whole oviducts by gentle squeezing, then the OF was isolated by two centrifugations (2000× *g*, 15 min then 12,000× *g*, 10 min) at 4 °C. The OF from 22 cows were pooled, assayed for protein concentration, divided into 15-µL aliquots and stored at −80 °C before used for incubation with embryos. The same pool of post-ovulatory OF was used for all embryo co-incubations.

### 4.2. Embryo In-Vitro Production and Incubation with OF

Bovine ovaries were collected from a local slaughterhouse and transported at 36 °C to the laboratory. Cumulus-oocyte complexes (COCs) were recovered using HEPES-buffered TCM-199 supplemented with 0.4 g/L bovine serum albumin (BSA) and 0.25% gentamicin. Groups of 30–60 COCs were matured in TCM-199 supplemented with 10 ng/mL EGF, 19 ng/mL IGF-1, 2.2 ng/mL FGF, 5 UI/mL hCG, 10 UI/mL PMSG, 4 µg/mL transferrin, 4 µg/mL insulin, 5 ng/mL sodium selenite, 1% PG-600, 90 µg/mL l-cysteine, 0.1 mM beta-mercaptoethanol, 75 µg/mL ascorbic acid, 720 µg/mL glycine, 0.1 mg/mL glutamine and 110 µg/mL pyruvate at 38.8 °C (5% CO2) for 22  h. After maturation, COCs (50 per well) were transferred in 250 µL of fertilization medium (Tyrode medium supplemented with 25 mM bicarbonate, 10 mM lactate, 1 mM pyruvate, 6 mg/mL fatty-acid free BSA, 10 µg/mL heparin and 40 µg/mL gentamycin). Motile spermatozoa were recovered by Percoll washing from one Normande bull (Evolution, Noyal-sur-Vilaine, France) and added to the fertilization medium (Day 0) at a final concentration of 10^6^ spermatozoa/mL for 22 h. At Day 1, all presumptive zygotes were cultured in 25 µL of synthetic oviductal fluid (SOF) medium [52] supplemented with 0.01% of polyvinyl alcohol (SOF-PVA) without any serum or protein supplementation, under mineral oil (M8410) at 38.8 °C with 5% CO_2_ and 5% O_2_. At Days 3 and 5, groups of 50 embryos of normal morphology (spherical embryos with blastomeres uniform in size, color and density) at the 4–6 cell and morula stages, respectively, were randomly allocated to incubation in 30 µL of OF at a final protein concentration of 37 mg/mL (OF-treated embryos) or in 30 µL of SOF-PVA (controls) for 6 h at 38.8 °C with 5% CO_2_ and 5% O_2_. After incubation, all embryos were washed three times in 20 mM Tris-HCl buffer (pH 6.8) supplemented with 8.9% sucrose (Tris-sucrose). Then, pools of 25 embryos were stored in 1.5-mL tubes at −80 °C for proteomics analysis. Four biological replicates were produced for 4–6 cells and morulas, i.e., 400 embryos for each stage (200 OF-treated and 200 controls).

### 4.3. Nanoliquid Chromatography Coupled with Tandem Mass Spectrometry (NanoLC-MS/MS)

For proteomics, pools of 25 embryos (OF-treated and controls) and one aliquot of OF were lysed in a 10 mM Trizma^®^ base supplemented with 4% sodium dodecyl sulfate (SDS) and 0.05% of a protease inhibitor cocktail for 15 min at ambient temperature. Protein concentrations were determined using a Nanodrop 2000/2000c^®^ (Thermo Scientific, Waltham, MA, USA). The samples were incubated for 5 min at 95 °C in Laemmli Buffer then protein lysates (4 µg for 4–6 cells, 6 µg for morulas, 15 µg for the OF) were briefly migrated on a home-made 0.75-mm thick 10% SDS-PAGE (50 V, 15 min) to get one band per sample. The gel bands were stained with BluePage^®^ overnight then proteins were in-gel digested with bovine trypsin, as previously described [5]. Salts were removed from samples using C18 SpinColumns (Harvard Apparatus, Les Ulis, France). The resulting peptide mixtures were separated on a 75 µm × 250 mm IonOpticks Aurora 2 C18 column (Ion Opticks Pty Ltd., Bundoora, Australia). A gradient of basic reversed-phase buffers (Buffer A: 0.1% formic acid, 98% H_2_O MilliQ, 2% acetonitrile; Buffer B: 0.1% formic acid, 100% acetonitrile) was run on a NanoElute HPLC System (Bruker Daltonik GmbH, Bremen, Germany) at a flow rate of 400 nL/min at 50 °C. The liquid chromatography (LC) run lasted for 120 min (2% to 15% of buffer B during 60 min; up to 25% at 90 min; up to 37% at 100 min; up to 95% at 110 min and finally 95% for 10 min to wash the column). The column was coupled online to a TIMS TOF Pro (Bruker Daltonik GmbH, Bremen, Germany) with a CaptiveSpray ion source (Bruker Daltonik). The temperature of the ion transfer capillary was set at 180 °C. Ions were accumulated for 114 ms, and mobility separation was achieved by ramping the entrance potential from −160 V to −20 V within 114 ms. The acquisition of the MS and MS/MS mass spectra was done with average resolutions of 60,000 and 50,000 full width at half maximum (mass range 100–1700 *m*/*z*), respectively. To enable the PASEF method, precursor *m*/*z* and mobility information was first derived from full scan TIMS-MS experiments (with a mass range of *m*/*z* 100–1700). The quadrupole isolation width was set to 2 and 3 Th and, for fragmentation, the collision energies varied between 31 and 52 eV depending on the precursor mass and charge. TIMS, MS operation and PASEF were controlled and synchronized using the control instrument software OtofControl 5.1 (Bruker Daltonik). LC-MS/MS data were acquired using the PASEF method with a total cycle time of 1.31 s, including 1 TIMS MS scan and 10 PASEF MS/MA scans. The 10 PASEF scans (100 ms each) containing, on average, 12 MS/MS scans per PASEF scan. Ion mobility-resolved mass spectra, nested ion mobility vs. *m*/*z* distributions, as well as summed fragment ion intensities were extracted from the raw data file with DataAnalysis 5.1 (Bruker Daltonik GmbH, Bremen, Germany).

### 4.4. Quantification of Proteins, Identification of Embryo-Interacting Proteins and Statistical Analysis

All proteins with more than two peptides identified were considered for protein quantification. Protein quantification was based on a label-free approach using spectral counting, as previously described [31]. Scaffold Q+ software (version 4.9, Proteome Software; www.proteomesoftware.com) was used using the Spectral Count quantitative module. Peptide identifications were accepted if they could be established with greater than 95.0% probability as specified by the Peptide Prophet algorithm [53]. Peptides were considered distinct if they differed in sequence. Protein identifications were accepted if they could be established with greater than 95.0% probability as specified by the Protein Prophet algorithm [54] and contained at least two identified peptides (false discovery rate (FDR) < 0.01%).

The normalization of spectra among the samples was realized in Scaffold by adjusting the sum of the selected quantitative values for all proteins within each MS sample to a common value, which was the average of the sums of all MS samples present in the experiment. This was achieved by applying a scaling factor for each sample to each protein or protein group. Thus, the numbers of the normalized weighted spectra (NWS) were tabulated using experiment-wide protein clusters.

Proteins were defined as embryo-interacting proteins originating in the OF if they met the following conditions: (i) detection at a minimum level of 5 NWS in the OF and (ii) detection at a minimum level of 5 NWS in OF-treated embryos with no detection in controls or significantly higher detection in OF-treated embryos than in controls after Student’s t-test with Benjamini–Hochberg correction (*p*-value < 0.05; fold-change > 2).

### 4.5. Functional Analyses of Interacting Proteins

To predict the secretion ways of embryo-interacting proteins, the online tools SignalP 5.0 (http://www.cbs.dtu.dk/services/SignalP/) and SecretomeP 2.0 (http://www.cbs.dtu.dk/services/SecretomeP/) were used. Proteins predicted as possessing a standard secretory signal peptide and a peptide cleavage site in their N-terminal sequence were considered as secreted in a conventional way. In addition, proteins predicted as being targeted to a non-classical pathway in the mammalian dataset of SecretomeP were considered as potentially undergoing non-conventional secretion. A cutoff NN-score value of 0.6 was applied. To go further in the non-conventional secretion pathway, we looked for the presence of the protein among those reported in bovine [9] and feline [30] oviductal EVs. The online tool Proteomaps 2.0 (https://bionic-vis.biologie.uni-greifswald.de) was used for the functional annotation of embryo-interacting proteins using NWS values and Gene IDs of interacting proteins, and the *Bos taurus* dataset.

### 4.6. Immunolocalization of ANXA1, OVGP1 and PYGL

By western blotting, the primary antibodies used gave one band at the expected molecular weight in bovine post-ovulatory oviduct epithelial cells and OF (Figure S2). For immunostaining, embryos of normal morphology at Day 3 were used. Embryos were incubated or not (controls) in OF and washed in Tris-sucrose as described above. Embryos were then fixed for 30 min in 4% paraformaldehyde at 35 °C then washed three times in PBS supplemented with 0.1% (*w*/*v*) BSA (PBS-BSA). For blocking, embryos were incubated for 40 min at ambient temperature in PBS-BSA supplemented with 10% (*v*/*v*) serum from the same host species as the secondary antibody (donkey for OVGP1 and PYGL; goat for ANXA1). After three washings in PBS-BSA, the embryos were incubated overnight at 4 °C with either anti-ANXA1, anti-OVGP1 (both at 1 µg/mL) or anti-PYGL (at 0.5 µg/mL) diluted in PBS-PSA. Isotypes at the same concentrations were used as negative controls. After two washings in PBS-BSA, embryos were incubated overnight at 4 °C in the secondary antibody diluted at 1/1000 in PBS-BSA. At the end of incubation, Hoechst 33,342 was added (10 µg/mL, 10 min) for nucleus staining. After three washings in PBS-BSA, embryos were mounted in PBS-BSA and immediately observed under confocal microscopy (Zeiss LSM 700, Carl Zeiss, Oberkochen, Germany). Two biological replicates were made for each antibody.

## Figures and Tables

**Table 1:** Proteins identified as interacting with embryos at the 4–6 cell stage.

Gene Name	Protein Name	Accession Number (UniprotKB)	Molecular Weight (kDa)	OF-Treated: Control Ratio
LZTFL1	Leucine zipper transcription factor-like protein 1	Q3ZBL4	35	T ^1^
NQO1	NAD(P)H quinone dehydrogenase 1	Q3ZBH2	31	T ^1^
ALDH1A2	Aldehyde dehydrogenase 1 family member A2	G3X6U1	57	T ^1^
HBA	Hemoglobin subunit alpha	P01966	15	T ^1^
ITGA2	Integrin alpha-2 (Fragment)	P53710	129	22
WARS	Tryptophan--tRNA ligase. cytoplasmic	P17248	54	8.7
RPS16	40S ribosomal protein S16	Q3T0X6	16	8.3
TARS	Threonine--tRNA ligase. cytoplasmic	Q3ZBV8	83	8.1
PFKL	ATP-dependent 6-phosphofructokinase. liver type	A1A4J1	85	4.9
RPL6	60S ribosomal protein L6	Q58DQ3	33	4.5
ALDH1A1	Retinal dehydrogenase 1	P48644	55	2.9
PPP2R1A	Alpha isoform of regulatory subunit A. protein phosphatase 2	Q32PI5	65	2.3
ANXA5	Annexin A5	P81287	36	2.3

^1^ Protein detected only in OF-treated embryos.

**Table 2:** Proteins identified as interacting with embryos at the morula stage.

Gene Name	Protein Name	Accession Number (UniprotKB)	Molecular Weight (kDa)	OF-Treated: Control Ratio
ACLY	ATP-citrate synthase	Q32PF2	120	T ^1^
ACO1	Cytoplasmic aconitate hydratase	Q0VCU1	98	T ^1^
PNP	Purine nucleoside phosphorylase	P55859	32	T ^1^
BLVRA	Biliverdin reductase A	A5D7K0	34	9.9
A2M	Alpha-2-macroglobulin	Q7SIH1	168	7
HBB	Hemoglobin subunit beta	P02070	16	6.7
APEX1	DNA-(apurinic or apyrimidinic site) lyase	P23196	36	5.8
TAGLN2	Transgelin-2	Q5E9F5	22	5.8
LGALS3	Galectin-3	A6QLZ0	28	5.3
LGALS9	Galectin-9	Q3MHZ8	39	4.3
TBCB	Tubulin-folding cofactor B	Q5E951	28	4.3
YWHAQ	14-3-3 protein theta	Q3SZI4	28	3
GLMN	Glomulin. FKBP associated protein	E1BA27	68	2.9
YWHAH	14-3-3 protein eta	P68509	28	2.9
MAP4	Microtubule-associated protein	P36225	112	2.6
TPP2	Tripeptidyl-peptidase 2	A5PK39	138	2.3
MYL6	Myosin light polypeptide 6	P60661	17	2.2
FKBP4	Peptidyl-prolyl cis-trans isomerase	Q9TRY0	51	2
HUWE1	HECT. UBA and WWE domain containing 1. E3 ubiquitin protein ligase	E1BNY9	482	3.4

^1^ Protein detected only in OF-treated embryos.

**Table 3:** Proteins identified as interacting with both the 4–6 cell and morula stages.

Gene Name	Protein Name	Accession Number (UniprotKB)	Molecular Weight (kDa)	4–6 Cell OF-Treated: Control Ratio	Morula OF-Treated: Control Ratio
ANXA8	Annexin A8	Q95L54	37	T ^1^	T ^1^
AGR2	Anterior gradient 2. protein disulphide isomerase family member	F1N3J3	20	T ^1^	T ^1^
CAPS	Calcyphosin	Q0VCC0	21	T ^1^	61
CD109	CD109 molecule	F1MPE1	161	T ^1^	T ^1^
CKB	Creatine kinase B-type	Q5EA61	43	T ^1^	4.1
CNDP2	Cytosolic non-specific dipeptidase	Q3ZC84	53	T ^1^	17
EPHX2	Epoxide hydrolase 2	F6QS88	63	T ^1^	T ^1^
PYGL	Glycogen phosphorylase. liver form	Q0VCM4	97	T ^1^	T ^1^
LRBA	LPS responsive beige-like anchor protein	E1BND6	316	T ^1^	8.2
DCXR	l-xylulose reductase	Q1JP75	26	T ^1^	T ^1^
OVGP1	Oviduct-specific glycoprotein	Q28042	60	T ^1^	71
PTGR2	Prostaglandin reductase 2	Q32L99	38	T ^1^	T ^1^
SEPTIN2	Septin-2	Q2NKY7	41	T ^1^	4.2
SARS	Serine--tRNA ligase. cytoplasmic	Q9GMB8	59	T ^1^	5
TPPP3	Tubulin polymerization-promoting protein family member 3	Q3ZCC8	19	T ^1^	T ^1^
ANXA1	Annexin A1	P46193	39	37	15
RNPEP	Uncharacterized protein	G3X743	72	9.6	4.6
FASN	Fatty acid synthase	Q71SP7	274	8.5	9.6
RNH1	Ribonuclease/angiogenin inhibitor 1	Q3SZN8	49	6.9	7.5
ANXA4	Annexin A4	P13214	36	5.4	6.5
SHTN1	Shootin 1	F1MUA7	63	5.3	4
ALDH9A1	4-trimethylaminobutyraldehyde dehydrogenase	Q2KJH9	54	4.2	2.9
ANXA2	Annexin A2	P04272	39	3.6	2.2
CLIC1	Chloride intracellular channel protein 1	Q5E9B7	27	2.1	3

^1^ Protein detected only in OF-treated embryos.

## Supplementary Materials

Supplementary Materials can be found at https://www.mdpi.com/1422-0067/21/2/466/s1.

## Abbreviations

OF	Oviductal fluid
EV	Extracellular vesicles
MS	Mass spectrometry
nanoLC-MS/MS	Nanoliquid chromatography coupled with tandem mass spectrometry
NWS	Normalized weighted spectra
