# Supplementary material for: Identification of 56 Proteins Involved in Embryo–Maternal Interactions in the Bovine Oviduct
Source: Int J Mol Sci. 2020 Jan 11;21(2):466. doi: 10.3390/ijms21020466 (PMC7013689; doi:10.3390/ijms21020466)

**Supplementary Figure 2.** Western blots of annexin A1 (ANXA1, 50 µg of proteins per lane), oviductin (OVGP1, 30 µg) and Glycogen phosphorylase, liver form (50 µg) in bovine post-ovulatory oviductal fluid (OF) and oviduct epithelial cells (BOEC).


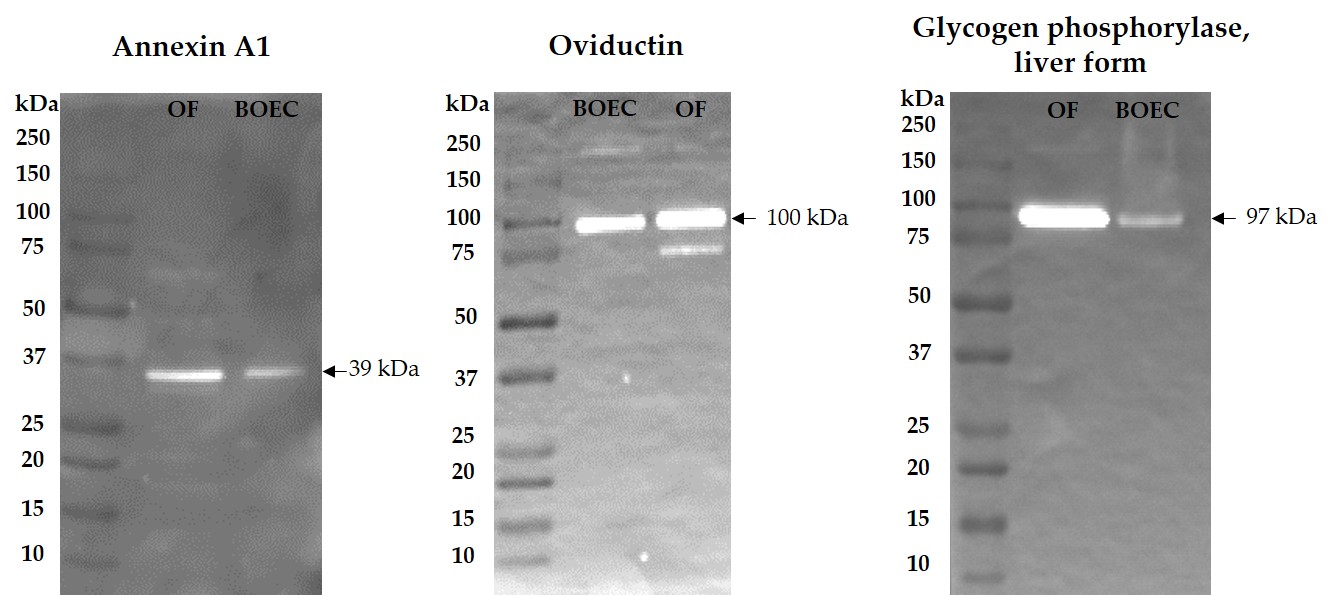

Supplement: Supplementary file 1 [file ijms-21-00466-s001.zip › Supplementary/Supplementary Figure 2.docx]
