# Supplementary material for: Identification of 56 Proteins Involved in Embryo–Maternal Interactions in the Bovine Oviduct
Source: Int J Mol Sci. 2020 Jan 11;21(2):466. doi: 10.3390/ijms21020466 (PMC7013689; doi:10.3390/ijms21020466)
Supplement: Supplementary file 1 [file ijms-21-00466-s001.zip › Supplementary/Supplementary Figure1.docx]

**Supplementary Figure 1**. Scatter plots showing the initial abundance of embryo-interacting proteins in the OF according to the abundance of the same proteins in OF-treated embryos at 4-6 cell (up) and morula (down) stages.
